# Eliminating leprosy?

**DOI:** 10.1002/ski2.16

**Published:** 2021-02-16

**Authors:** A. Gohar

**Affiliations:** ^1^ Departments of Dermatology and Genitourinary Medicine Hosary Medical Charity Centre Giza Egypt

I read with great interest, the study by Mowla MR et al.^1^

Unfortunately, the World Health Organization (WHO)'s rhetoric in the relevant programme has led to the impression, at least in some quarters, that leprosy no longer exists. Furthermore, it is likely that neither funders nor young researchers are attracted to an officially ‘eliminated’ disease—even if it is still ubiquitous.^2^

A footnote to the relevant WHO resolution explained that elimination was to be defined in this context as a reduction in prevalence below 1 per 10 000.^2^ However, this has been misleading.^2^ Leprosy still exists and as the authors stated the disease continues to be significant cause of peripheral neuropathy, deformity, disability and disfigurement in some countries.^1^

## CONFLICT OF INTEREST

Author has no conflict of interest to declare.
